# Comparison of Hospital Volume and Risk-Standardized Mortality Rate as a Proxy for Hospital Quality in Complex Oncologic Hepatopancreatobiliary Surgery

**DOI:** 10.1245/s10434-024-15361-2

**Published:** 2024-05-03

**Authors:** William T. Julian, Mohamedraed Elshami, John B. Ammori, Jeffrey M. Hardacre, Lee M. Ocuin

**Affiliations:** 1 Case Western Reserve University School of Medicine, Cleveland, OH USA; 2 Department of Surgery, Division of Surgical Oncology, University Hospitals Cleveland Medical Center, Cleveland, OH USA

**Keywords:** Risk-standardized mortality rate, Postoperative, Hospital volume, Hospital region, Hepatopancreatobiliary surgery

## Abstract

**Background:**

Centralization of hepatopancreatobiliary procedures to more experienced centers has been recommended but remains controversial. Hospital volume and risk-stratified mortality rates (RSMR) are metrics for interhospital comparison. We compared facility operative volume with facility RSMR as a proxy for hospital quality.

**Patients and Methods:**

Patients who underwent surgery for liver (LC), biliary tract (BTC), and pancreatic (PDAC) cancer were identified in the National Cancer Database (2004–2018). Hierarchical logistic regression was used to create facility-specific models for RSMR. Volume (high versus low) was determined by quintile. Performance (high versus low) was determined by RSMR tercile. Primary outcomes included median facility RSMR and RSMR distributions. Volume- and RSMR-based redistribution was simulated and compared for reductions in 90-day mortality.

**Results:**

A total of 106,217 patients treated at 1282 facilities were included; 17,695 had LC, 23,075 had BTC, and 65,447 had PDAC. High-volume centers (HVC) had lower RSMR compared with medium-volume centers and low-volume centers for LC, BTC, and PDAC (all *p *< 0.001). High-performance centers (HPC) had lower RSMR compared with medium-performance centers and low-performance centers for LC, BTC, and PDAC (all *p *< 0.001). Volume-based redistribution required 16.0 patients for LC, 11.2 for BTC, and 14.9 for PDAC reassigned to 15, 22, and 20 centers, respectively, per life saved within each US census region. RSMR-based redistribution required 4.7 patients for LC, 4.2 for BTC, and 4.9 for PDAC reassigned to 316, 403, and 418 centers, respectively, per life saved within each US census region.

**Conclusions:**

HVC and HPC have the lowest overall and risk-standardized 90-day mortality after oncologic hepatopancreatobiliary procedures, but RSMR may outperform volume as a measure of hospital quality.

**Supplementary Information:**

The online version contains supplementary material available at 10.1245/s10434-024-15361-2.

Resection remains a necessary component in multimodal management of resectable hepatopancreatobiliary cancers in medically appropriate patients. Surgical procedures of the liver, biliary tract, and pancreas are high-acuity and high-complexity operations associated with some of the highest morbidity and mortality rates as compared with other common gastrointestinal oncologic procedures, and interhospital differences in outcomes continue to persist.^1–5^

Volume-based redistribution to higher-volume centers is a possible measure to improve patient outcomes.^4,6,7^ Throughout the Canadian and European healthcare systems with universal coverage, centralization has been implemented with reported decreases in postoperative mortality for select cancers.^8^ In the USA, barriers to volume-based redistribution exist, including the paucity of high-volume surgical centers for complex oncological surgery and the heterogeneity of outcomes at different centers.^9,10^ Logistical challenges exist given the absence of a single-payer system and the lack of programs to assist with financial- and transportation-related toxicity associated with cancer care.^11–16^

A direct comparison of traditional outcome measures between centers is complicated by factors including case mix, case complexity, and clinical and sociodemographic differences in baseline patient populations.^17^ The risk-standardized mortality rate (RSMR) is a healthcare indicator utilized for comparison of regions and hospitals while adjusting for these potential baseline differences between centers.^18^ RSMR has also been used as an alternative proxy for hospital quality.^19^ RSMR is calculated as the ratio of a hospital’s predicted mortality to its expected mortality rate using hierarchical logistic regression analysis.^20^

In this study, we utilize a large, administrative dataset to analyze postoperative mortality following hepatopancreatobiliary operations stratified by tumor location, hospital operative volume, and RSMR. A redistribution of patients was performed using both a volume- or RSMR-based approach to estimate the effect of patient redistribution to high-volume or higher-performing centers on 90-day postoperative mortality.

## Patients and Methods

### Institutional Assurances

This study was exempt from institutional review board (IRB) approval at University Hospitals Cleveland Medical Center as it utilized deidentified patient data from the National Cancer Database (NCDB).

### Data Source

The NCDB is maintained by the American Cancer Society and the American College of Surgeons. The NCDB includes patient data from more than 1500 programs accredited by the Commission on Cancer throughout the USA, and contains data for approximately 70% of all malignancies diagnosed in the USA.^21^

### Patient Selection

Patients who underwent resection of hepatopancreatobiliary cancer [liver cancer (LC), biliary tract cancer (BTC), and pancreatic cancer (PDAC)] within the NCDB between 2004 and 2018 were selected and stratified on the basis of tumor location according to procedure-specific codes in the data dictionary.^21^ Patients were identified in respective participant user files using International Classification of Diseases for Oncology histological codes (Supplementary Table 1). Procedure and codes included in the analysis are presented in Supplementary Table 1. Wedge/segmental resection for LC and BTC were classified as minor hepatectomy, whereas lobectomy, extended lobectomy, and hepatectomy were classified as major hepatectomy. The flowchart for patient selection is demonstrated in Supplementary Fig. 1.

### Variables of Interest

Patient characteristics included age, sex, race, Charlson–Deyo score, insurance status, income, and education level. Treatment included receipt of chemotherapy and receipt of radiotherapy. Operative outcomes included 30- and 90-day mortality, length of stay, and 30-day readmission rates. Pathologic outcomes included margin status, number of lymph nodes analyzed, and nodal positivity. Facility characteristics included hospital type (academic versus non-academic), distance to treating facility, and median annual operative hospital volume stratified by tumor location. Academic facilities were defined as an academic/research institution or integrated network program.^21^ The US census region of each hospital was captured for our redistribution model.

### Outcomes Measures

The primary outcome was median facility RSMR and RSMR distributions, stratified by facility operative volume and tumor location. The secondary outcome was a redistribution analysis using a volume-based or RSMR-based approach.

### Statistical Analysis

Hierarchical logistic regression was performed by the facility to model risk-stratified mortality rates and was performed using the lme4 package using R statistical software (R Foundation for Statistical Computing, Vienna, Austria). The model included patient age, sex, and Charlson–Deyo score to predict patient mortality at 90 days, stratified by tumor location.^22^ Each facility received a specific model predicting outcome that could be compared with the overall average model (expected). RSMR values were calculated as the ratio of predicted outcome to expected outcome for each facility.

Hospitals were stratified by operative volume percentile. The top quintile comprised high-volume centers (HVC) and the bottom quintile comprised low-volume centers (LVC); the middle three quintiles comprised medium-volume centers (MVC) as performed in prior analyses.^18^ Hospitals were also stratified by facility RSMR using terciles to minimize differences in patient numbers per group and facilitate redistribution analysis. The lowest tercile comprised high-performance centers (HPC), the highest tercile comprised low-performance centers (LPC), and the middle tercile comprised medium-performance centers (MPC). Outcome data were calculated for HVC, MVC, and LVC, as well as HPC, MPC, and LPC.

Redistribution analysis was performed using a volume-based approach and compared with a RSMR-based approach. Patients treated at LVC or MVC were randomly reassigned to HVC (volume based) and patients treated at LPC were subjected to reassignment to HPC (RSMR based) within the same US census region. Patient mortality probabilities were calculated before and after reassignment using the original hierarchical logistic regression models per facility. All simulations were performed 1000 times.

All statistical analysis was performed using R. The Kruskal–Wallis test was used for comparison of quantitative variables of interest with alpha = 0.05. The pairwise Wilcoxon test was used for ad hoc testing of quantitative variables. Chi-squared testing was used for comparison of categorical variables with alpha = 0.05. The pairwise proportion test was used for ad hoc testing of categorical variables.

## Results

### Volume-Based Analysis: Baseline Demographic and Clinical Characteristics

A total of 106,217 patients who underwent operative management of LC, BTC, and PDAC at 1282 facilities were included in the analysis. Clinical and demographic characteristics are reported in Supplementary Table 2.

A total of 17,695 patients were treated for LC, of whom 19.9% were treated at HVC, 59.9% at MVC, and 20.1% at LVC. Median annual facility volumes were 0.2 at LVC, 2.7 at MVC, and 12.9 at HVC (Supplementary Fig. 2A). A total of 15 facilities were HVC, 197 were MVC, and 734 were LVC.

A total of 23,075 patients were treated for BTC, of whom 20.3% were treated at HVC, 59.7% at MVC, and 20.1% at LVC (Supplementary Table 2). A total of 22 facilities were HVC, 338 were MVC, and 848 were LVC. Median annual facility volume was 0.3 at LVC, 1.9 at MVC, and 11.9 at HVC (Supplementary Fig. 2B).

A total of 65,447 patients were treated for PDAC, of whom 20.3% were treated at HVC, 59.7% at MVC, and 20.0% at LVC (Supplementary Table 2). A total of 20 facilities were HVC, 318 were MVC, and 915 were LVC. Median annual facility volume was 0.7 at LVC, 5.9 at MVC, and 36.2 at HVC (Supplementary Fig. 2C).

### Volume-Based Analysis: Postoperative Outcomes

**Fig. 1:**
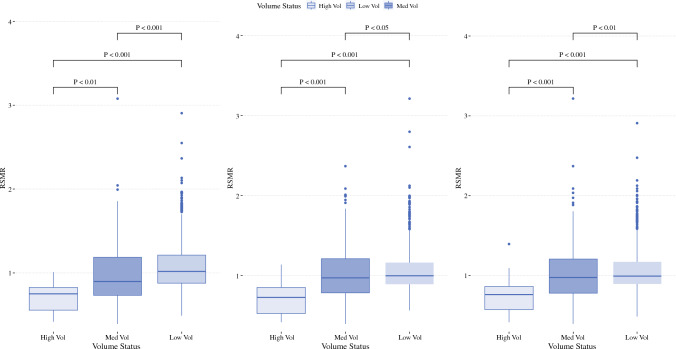
Median RSMR by volume group; **A** liver cancer, **B** BTC, and **C** pancreatic cancer

**Fig. 2:**
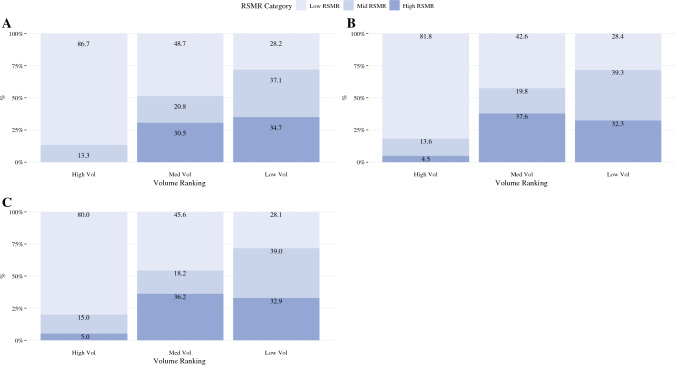
RSMR distributions by volume group; **A** liver cancer, **B** BTC, and **C** pancreatic cancer

Pathologic and postoperative outcomes stratified by hospital volume and tumor location are reported in Table 1A. For patients with LC, the 30-day mortality rate was ~ threefold higher at LVC versus HVC. Similarly, the 90-day mortality was ~ twofold higher at LVC versus HVC (all *p *< 0.001). The median RSMR was 0.75 at HVC, 0.90 at MVC, and 1.02 at LVC (*p *< 0.001; Fig. 1A). Most HVC (86.7%) were classified as HPC, compared with 48.7% of MVC and 28.2% of LVC (*p* < 0.001; Fig. 2A).

**Table 1:** Pathologic and postoperative outcomes, stratified by (A) volume and tumor location and (B) RSMR and tumor location

Characteristics	Liver cancer				BTC				Pancreatic cancer			
	Low volume	Medium volume	High volume	*p* value	Low volume	Medium volume	High volume	*p *value	Low volume	Medium volume	High volume	*p* value
*(A)*												
Lymph nodes removed (median, IQR)	0 (0)	0 (0)	0 (0)	0.062	2 (9)	5 (12)	7 (15)	< 0.001	13 (12)	15 (12)	17 (13)	< 0.001
Margin positivity, *n* (%)	411 (11.5)	898 (8.5)	230 (6.5)	< 0.001	1160 (25.1)	2898 (21)	802 (17.2)	< 0.001	3181 (24.3)	8619 (22.1)	2601 (19.6)	< 0.001
Nodal positivity	31 (2.2)	88 (1.9)	38 (2.4)	0.482	1131 (43.3)	4086 (44.7)	1529 (46.7)	0.024	7291 (65.6)	21738 (63.7)	7612 (64.8)	< 0.001
Chemotherapy	410 (11.5)	1309 (12.3)	550 (15.6)	< 0.001	2185 (47.2)	6133 (44.5)	2074 (44.4)	< 0.001	9621 (73.4)	27053 (69.3)	9452 (71.2)	< 0.001
Radiation	91 (2.6)	321 (3)	101 (2.9)	< 0.001	1212 (26.2)	3014 (21.9)	881 (18.8)	< 0.001	5552 (42.4)	12978 (33.2)	4228 (31.8)	< 0.001
Length of stay (median, IQR)	6 (5)	6 (4)	6 (4)	0.045	6 (8)	8 (7)	8 (6)	< 0.001	8 (8)	8 (7)	8 (5)	< 0.001
30-day readmission,* n* (%)	226 (6.6)	634 (6.1)	260 (7.5)	< 0.001	310 (7)	1310 (9.7)	377 (8.2)	< 0.001	932 (7.6)	3586 (9.4)	1220 (9.3)	< 0.001
30-day mortality	256 (7.2)	484 (4.6)	100 (2.8)	< 0.001	312 (6.7)	681 (4.9)	154 (3.3)	< 0.001	541 (4.1)	1213 (3.1)	276 (2.1)	< 0.001
90-day mortality	392 (11)	852 (8)	205 (5.8)	< 0.001	614 (13.3)	1331 (9.7)	313 (6.7)	< 0.001	1020 (7.8)	2584 (6.6)	639 (4.8)	< 0.001
Median RSMR	1.02	0.90	0.75	< 0.001	1.00	0.97	0.72	< 0.001	0.99	0.97	0.76	< 0.001

Characteristics	Liver				BTC				Pancreas			
	HPC	MPC	LPC	*p* value	HPC	MPC	LPC	*p* value	HPC	MPC	LPC	*p* value
*(B)*												
Lymph nodes removed (median, IQR)	0 (0)	0 (0)	0 (0)	0.006	5 (13)	4 (13)	3 (11)	< 0.001	16 (13)	15 (13)	12 (11)	< 0.001
Margin positivity, *n* (%)	755 (7.7)	358 (9.72)	426 (10.12)	< 0.001	2382 (19.37)	1095 (23.15)	1383 (22.87)	< 0.001	7429 (19.98)	2811 (23.57)	4161 (25.45)	< 0.001
Nodal positivity	97 (2.25)	25 (1.67)	35 (2)	0.389	3724 (45.02)	1398 (45.6)	1624 (44.03)	0.413	21153 (64.03)	6567 (65.99)	8921 (63.74)	< 0.001
Chemotherapy	1388 (14.16)	484 (13.13)	397 (9.43)	< 0.001	5889 (47.89)	2063 (43.61)	2440 (40.36)	< 0.001	27288 (73.41)	8329 (69.84)	10509 (64.28)	< 0.001
Radiation	330 (3.37)	95 (2.58)	88 (2.09)	< 0.001	2749 (22.35)	1010 (21.35)	1348 (22.3)	0.036	13163 (35.41)	4036 (33.84)	5559 (34)	< 0.001
Length of stay (median, IQR)	5 (4)	6 (4)	6 (5)	< 0.001	7 (7)	7 (8)	7 (9)	0.004	8 (6)	8 (7)	9 (8)	< 0.001
30-day readmission, *n* (%)	635 (6.63)	201 (5.62)	284 (6.85)	< 0.001	1058 (8.83)	346 (7.53)	593 (10)	< 0.001	3174 (8.8)	904 (7.81)	1660 (10.43)	< 0.001
30-day mortality	281 (2.87)	184 (4.99)	375 (8.91)	< 0.001	376 (3.06)	247 (5.22)	524 (8.67)	< 0.001	664 (1.79)	352 (2.95)	1014 (6.2)	< 0.001
90-day mortality	534 (5.45)	338 (9.17)	577 (13.71)	< 0.001	758 (6.16)	506 (10.7)	994 (16.44)	< 0.001	1504 (4.05)	784 (6.57)	1955 (11.96)	< 0.001
Median RSMR	0.79	1.00	1.32	< 0.001	0.81	0.99	1.29	< 0.001	0.81	0.98	1.29	< 0.001

For patients with BTC, the 30-day and 90-day mortality rates were ~ twofold higher at LVC versus HVC (all *p *< 0.001). The median RSMR was 0.72 at HVC, 0.97 at MVC, and 1.0 at LVC (*p *< 0.001; Fig. 1B). Most HVC (81.8%) were classified as HPC, compared with 42.3% of MVC and 28.5% of LVC (*p *< 0.001; Fig. 2B).

For patients with PDAC, the 30-day and 90-day mortality rates were ~ twofold higher at LVC versus HVC (all *p *< 0.001). Median RSMR values were 0.76 at HVC, 0.97 at MVC, and 0.99 at LVC (*p *< 0.001; Fig. 1C). Most HVC (80.0%) were classified as HPC, compared with 45.3% of MVC and 28.2% of LVC (*p *< 0.001; Fig. 2C).

### RSMR-Based Analysis

When stratified by RSMR tercile, 9811 patients with LC were treated at HPC, 3762 at MPC, and 4122 at LPC (Supplementary Table 3). A total of 316 facilities were HPC, 315 were MPC, and 315 were LPC. The median annual operative volume for LC was 0.5 (IQR 2.07) at HPC, 0.2 (IQR 0.53) at MPC, and 0.33 (IQR 0.8) at LPC (Supplementary Fig. 3A). The 30-day and 90-day mortality rate was lowest at HPC (Table 1B). The median facility RSMR was 0.79 at HPC, 1.00 at MPC, and 1.32 at LPC (*p* < 0.001).

A total of 12,312 patients with BTC were treated at HPC, 4672 at MPC, and 6091 at LPC (Supplementary Table 3). A total of 403 centers were HPC, 403 centers were MPC, and 402 centers were LPC. The median annual facility operative volume was 0.67 at HPC, 0.27 at MPC, and 0.6 at LPC (Supplementary Fig. 3B). The 30-day and 90-day mortality rates were lowest at HPC (Table 1B). The 90-day mortality rate was 6.2% at HPC, 10.9% at MPC, and 16.3% at LPC (*p *< 0.001). The median facility RSMR was 0.81 at HPC, 0.99 at MPC, and 1.29 at LPC (*p *< 0.001).

A total of 37,179 patients with PDAC were treated at HPC, 11,815 at MPC, and 15,453 at LPC (Supplementary Table 3). A total of 418 centers were HPC, 418 centers were MPC, and 417 centers were LPC. The median annual operative volume was 1.9 at HPC, 0.6 at MPC, and 1.47 at LPC (Supplementary Fig. 3C). The 30-day mortality and 90-day mortality rates were lowest at HPC (Table 1B). The 90-day mortality rate was 4.0% at HPC, 6.5% at MPC, and 12.0% at LPC (*p *< 0.001). The median facility RSMR was 0.81 at HPC, 0.98 at MPC, and 1.29 at LPC (*p *< 0.001).

### Case Mix Analysis

**Fig. 3:**
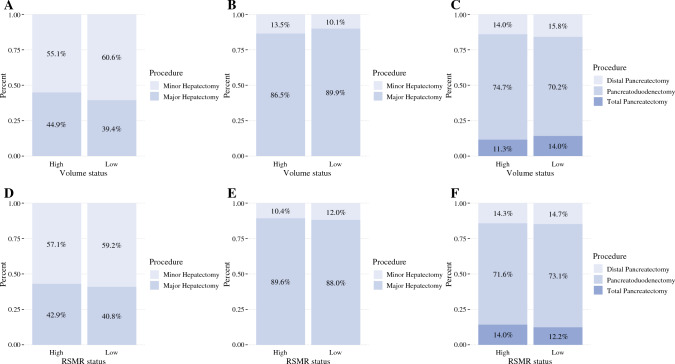
Case distribution by volume status; **A** liver cancer, **B** BTC, and **C** pancreatic cancer; case distribution by RSMR status; **D** liver cancer, **E** BTC, and **F** pancreatic cancer

Volume-based analysis of the case mix between HVC and LVC treating patients with LC demonstrated that LVC performed more minor hepatectomies and HVC performed more major hepatectomies (all *p *< 0.001; Fig. 3A). For patients with BTC, HVC performed more minor hepatectomies (and fewer major hepatectomies as compared with LVC (all *p *< 0.001; Fig. 3B). For patients with PDAC, HVC performed pancreatoduodenectomy more frequently and distal/total pancreatectomy less frequently than LVC (all *p *< 0.001; Fig. 3C).

For patients with LC, 90-day mortality rates were nearly twofold higher after minor or major hepatectomy at LVC as compared with HVC (all *p *< 0.001; Supplementary Fig. 4A). For patients with BTC, 90-day mortality rates were ~ 1.3–2-fold higher after minor or major hepatectomy at LVC compared with HVC, respectively (all *p *< 0.001; Supplementary Fig. 4B). For patients with PDAC, 90-day mortality rates at LVC were ~ twofold higher after distal pancreatectomy, ~ 1.6-fold higher after pancreatoduodenectomy, and ~ 1.5-fold higher after total pancreatectomy compared with HVC (all *p *< 0.001; Supplementary Fig. 4C).

RSMR-based analysis of the case mix between HPC and LPC treating patients with LC demonstrated that HPC performed more minor hepatectomies and fewer major hepatectomies compared with LPC (all *p *< 0.001; Fig. 3D). For patients with BTC, HPC performed more minor hepatectomies and fewer major hepatectomies compared with LPC (all *p *< 0.001; Fig. 3E). For patients with PDAC, HPC performed pancreatoduodenectomy more frequently and total pancreatectomy less frequently than LPC (all *p *< 0.001; Fig. 3F); there was no difference in the rate of distal pancreatectomy between HPC and LPC (14.7% versus 14.3%, *p *= 0.25).

For patients with LC, case-based 90-day mortality rates at LPC were ~ threefold higher after minor hepatectomy and ~ twofold higher after major hepatectomy compared with HPC (all *p *< 0.001; Supplementary Fig. 4D). For patients with BTC, 90-day mortality rates at LPC were nearly ~ threefold higher after minor or major hepatectomy compared with HPC (all *p *< 0.001; Supplementary Fig. 4E). For patients with PDAC, 90-day mortality rates at LPC were ~ threefold higher after any pancreatectomy compared with HPC (all *p *< 0.001; Supplementary Fig. 4F).

### Redistribution Analysis

Redistribution of patients using the volume-based approach resulted in reassignment of 14,168 patients with LC, 18,399 patients with BTC, and 52,163 patients with PDAC from a LVC or MVC to a HVC (Table 2 and Supplementary Table 4), resulting in 885 fewer 90-day mortalities in patients with LC, 1642 fewer in patients with BTC, and 3494 fewer in patients with PDAC. To prevent one 90-day postoperative death, 16 patients with LC, 11 patients with BTC, and 15 patients with PDAC required reassignment.

**Table 2:** Redistribution analysis by volume and RSMR

Characteristics	Volume model			RSMR model		
	Liver	BTC	Pancreas	Liver	BTC	Pancreas
Patients moved	14,168	18,399	52,163	4104	6046	16,349
Lives saved	885	1642	3494	874	1431	3332
Patients needed to move per life saved	16.0	11.2	14.9	4.7	4.2	4.9
Receiving centers	15	22	20	316	403	418

Redistribution of patients using the RSMR-based approach resulted in reassignment of 4104 patients with LC, 6046 patients with BTC, and 16,349 patients with PDAC from a LPC to a HPC (Table 2 and Supplementary Table 5), resulting in 874 fewer 90-day mortalities in patients with LC, 1431 fewer in patients with BTC, and 3332 fewer in patients with PDAC. To prevent one 90-day postoperative death, five patients with LC, five patients with BTC, and five patients with PDAC required reassignment. The RSMR-based approach resulted in fewer patients moved to prevent one death for all types of cancer (*p *< 0.001).

## Discussion

Volume-based centralization may address heterogenous interfacility outcomes in complex oncologic hepatopancreatobiliary surgery, however, direct comparisons between centers remains difficult given differences in patient population and case mix/complexity. RSMR is an alternative and possibly more effective method for evaluating hospital performance.^18^ In the current study, we report that HVC had lower RSMR, higher percentage of low-RSMR facilities, and lower 90-day mortality compared with MVC and LVC in patients with LC, BTC, or PDAC. RSMR-based patient redistribution appeared to be more effective and feasible than volume-based redistribution, saving a similar number of lives as volume-based redistribution while distributing patients to a higher number of facilities.

The relationship between hospital volume and patient mortality is well established, with previous studies demonstrating lower mortality with increasing surgical volume within breast, colorectal, and bariatric procedures.^23–27^ Our analysis demonstrates that lower 30- and 90-day mortality correlates with lower median facility RSMR across cancer types studied. Given differences in clinical and demographic features in patients as well as procedural variables between hospitals, median RSMR may provide a more accurate comparison of high-volume, medium-volume, and low-volume centers.

Studies suggest that RSMR may be more effective at identifying high-performing centers. Baum et al. analyzed patients undergoing surgical treatment for esophageal, lung, stomach, pancreas, and colon cancer in Germany, and reported that RSMR was a better proxy for hospital quality, with centralization of facility based on RSMR requiring fewer patients to prevent 1 death for both individual cancer types and overall (32 versus 47), with RSMR-based redistribution saving more patients (955 versus 663) than a volume-based approach with fewer overall patients moved (30,253 versus 31,155).^18^ Chalif et al.^28^ reported that RSMR-based redistribution for glioblastoma saved 31.6% more lives with fewer patients needed to move per life saved (36 versus 46) compared with realignment based on volume alone. In our study, RSMR-based redistribution was more efficient in reducing patient mortality per patient moved, requiring 2–3-fold fewer patients to be moved as compared with volume-based redistribution while achieving the same reduction in mortality. In addition, the RSMR-based redistribution placed patients in more recipient centers, suggesting that RSMR-based redistribution could limit the logistic difficulties associated with redistribution. These findings are consistent with the study by Baum et al.^18^ A strict focus on hospital volume may overlook high-performing, lower-volume centers and fail to identify some low-performing centers. As we report, nearly one-third of LVC and half of MVC are HPC across the three cancers studied, while ~ 10–20% of HVC are lower-performing centers based on RSMR. Interestingly, no HVC treating liver cancer was classified as an LPC, compared with 4.5% of centers treating BTC and 5% of centers treating PDAC, suggesting that the relationship between volume and RSMR may differ for liver cancer compared with BTC and PDAC; in other words, volume and outcomes/RSMR may be more tightly associated in facilities treating patients with liver cancer as compared with those treating patients with BTC or PDAC. Additionally, our analysis of 90-day mortality rates stratified by case type demonstrated higher mortality rates at LVC and LPC compared with HVC and HPC across all procedures. These results are novel, and may support earlier studies that demonstrated that adjusting for case mix did not significantly impact analysis of operative mortality when investigating facility surgical volumes.^29^

The origin of differences in facility RSMR must be examined and its limitations discussed. The hierarchical logistic regression model used in this analysis calculated a hospital specific intercept, which served to approximate hospital quality. The NCDB does not provide facility-specific factors that may contribute to observed differences in our primary and secondary outcomes. The lack of facility-specific factors may decrease the effect size of a volume-based or RSMR-based redistribution, as HPC-specific protocols may not be generalizable to other treating facilities. Further institutional-based analyses could identify factors contributing to hospital performance, allowing for adoption and implementation in other hospitals, which could reduce the need for patient redistribution.

We acknowledge that redistribution may present logistical challenges, including those related to patient resources or lack of capacity at receiving facilities to manage the increased patient volume. Unfortunately, the NCDB only provides distance to the actual treating facility for a given patient and does not provide detailed geographic information for specific facilities or patients. There is no way to calculate distance to an alternative treating facility following redistribution. However, a volume-based reassignment would more likely create an undue burden on patients due to increased travel distances and worsen patient outcomes by reducing access to care to a smaller number of centers. The larger pool of available HPC using a RSMR-based approach may lessen the travel burden of redistribution on patients and ease hospital capacity constraints at receiving centers. Patients treated at HVC and HPC were less likely to come from low-income areas, more likely to have private insurance, and live further from the treatment facility, suggesting the possibility of selection bias, which may influence the association between redistribution on patient mortality. RSMR values alongside traditional outcome measures may account for differences in patient populations in comparing hospital facilities, but it may not address all demographic differences in populations undergoing treatment at different facilities. An individualized approach toward facility selection considering all patient demographic factors would be optimal, in which RSMR could facilitate a pragmatic discussion of benefits and risks on a case-by-case basis.

In conclusion, RSMR may be a more useful indicator of hospital quality compared with facility volume. Given the current discussion of procedural centralization, these findings are potentially impactful as a RSMR-based approach would allow for centers of various sizes in a wider geographical area to serve as preferential centers for patient care. This study suggests that facility-specific factors rather than patient-specific or case-specific factors drive observed differences in 90-day postoperative mortality following index HPB operations. Identification of more granular facility-specific factors contributing to reduced RSMR at high-performance centers could be generalized to other facilities to improve patient care on a national level across more hospitals.

### Supplementary Information

Below is the link to the electronic supplementary material.

Supplementary file1 (DOCX 44 kb)

Supplementary file2 (TIF 1246 kb)

Supplementary file3 (TIF 44 kb)

Supplementary file4 (TIF 55 kb)

Supplementary file5 (TIF 465 kb)
