# Supplementary material for: Comparison of Hospital Volume and Risk-Standardized Mortality Rate as a Proxy for Hospital Quality in Complex Oncologic Hepatopancreatobiliary Surgery
Source: Ann Surg Oncol. 2024 May 3;31(8):4922–30. doi: 10.1245/s10434-024-15361-2 (PMC11236847; doi:10.1245/s10434-024-15361-2)
Supplement: Supplementary file 1 — Supplementary file1 (DOCX 44 kb) [file 10434_2024_15361_MOESM1_ESM.docx]

**Supplementary Figure 1.** Patient selection flowchart for the study cohorts.

**Supplementary Figure 2.** Facility operative volume by volume status, A) liver cancer, B) BTC, and C) pancreatic cancer.

**Supplementary Figure 3.** Facility operative volume by RSMR status, A) liver cancer, B) BTC, and C) pancreatic cancer.

**Supplementary Figure 4.** 90-day mortality by operation and volume/RSMR status, A) liver cancer, B) BTC, and C) pancreatic cancer. 90-day mortality by operation and RSMR status, D) liver cancer, E) BTC, and F) pancreatic cancer.

| **Supplementary Table 1. Procedure codes included in the analysis by tumor type.** | | | | | |
| --- | --- | --- | --- | --- | --- |
| **Liver** | **Definition** | **BTC** | **Definition** | **Pancreas** | **Definition** |
| 20 | Wedge or segmental resection, NOS | 20 | Wedge or segmental resection, NOS | 30 | Partial pancreatectomy, NOS |
| 21 | Wedge resection | 21 | Wedge resection | 35 | Local or partial pancreatectomy and duodenectomy |
| 22 | Segmental rection, NOS | 22 | Segmental rection, NOS | 36 | Local or partial pancreatectomy and duodenectomy, without distal/partial gastrectomy |
| 23 | Segmental resection, one | 23 | Segmental resection, one | 37 | Local or partial pancreatectomy and duodenectomy with partial gastrectomy (Whipple) |
| 24 | Segmental resection, two | 24 | Segmental resection, two | 40 | Total pancreatectomy |
| 25 | Segmental resection, three | 25 | Segmental resection, three | 60 | Total pancreatectomy and subtotal gastrectomy or duodenectomy |
| 26 | Segmental resection and local tumor destruction | 26 | Segmental resection and local tumor destruction | 70 | Extended pancreatoduodenectomy |
| 30 | Lobectomy, NOS | 30 | Lobectomy, NOS |  |  |
| 36 | Right lobectomy | 36 | Right lobectomy |  |  |
| 37 | Left Lobectomy | 37 | Left Lobectomy |  |  |
| 38 | Lobectomy and local tumor destruction | 38 | Lobectomy and local tumor destruction |  |  |
| 50 | Extended lobectomy (NOS) | 50 | Extended lobectomy (NOS) |  |  |
| 51 | Right lobectomy, extended | 51 | Right lobectomy, extended |  |  |
| 52 | Left Lobectomy, extended | 52 | Left Lobectomy, extended |  |  |
| 59 | Extended lobectomy and local tumor destruction | 59 | Extended lobectomy and local tumor destruction |  |  |
| 60 | Hepatectomy, NOS | 60 | Hepatectomy, NOS |  |  |
| 65 | Excision of bile duct |  |  |  |  |
| 66 | Excision of bile duct and partial hepatectomy |  |  |  |  |
| **ICD-O histology codes included in analysis by tumor type.** | | | | | |
| **Liver** | **Definition** | **BTC** | **Definition** | **Pancreas** | **Definition** |
| 8170-8175 | Hepatocellular carcinoma | 8000, 8010, 8140, 8160, 8162 | Intrahepatic cholangiocarcinoma, extrahepatic cholangiocarcinoma, gallbladder adenocarcinoma | 8140, 8500 | Pancreatic adenocarcinoma |

| **Supplementary Table 2.** Baseline clinical and demographic characteristics, stratified by volume and tumor location. | | | | | | | | | |
| --- | --- | --- | --- | --- | --- | --- | --- | --- | --- |
| Characteristics | Liver Cancer | | | BTC | | | Pancreatic Cancer | | |
|  | Low Volume | Medium Volume | High Volume | Low Volume | Medium Volume | High Volume | Low Volume | Medium Volume | High Volume |
| Total Patients | 3561 | 10607 | 3527 | 4629 | 13770 | 4676 | 13099 | 39064 | 13284 |
| Age (median, IQR) | 67 (15) | 65 (14) | 64 (14) | 69 (17) | 67 (16) | 67 (15) | 67 (15) | 67 (14) | 67 (15) |
| Male sex, n (%) | 2451 (68.8) | 7588 (71.5) | 2585 (73.3) | 2042 (44.1) | 6807 (49.4) | 2434 (52.1) | 6740 (51.5) | 19861 (50.8) | 6813 (51.3) |
| Race |  |  |  |  |  |  |  |  |  |
| White | 2258 (63.4) | 6225 (58.7) | 1989 (56.4) | 3380 (73) | 9781 (71) | 3661 (78.3) | 10143 (77.4) | 30316 (77.6) | 11185 (84.2) |
| Black | 509 (14.3) | 1640 (15.5) | 442 (12.5) | 413 (8.9) | 1178 (8.6) | 313 (6.7) | 1118 (8.5) | 3764 (9.6) | 1017 (7.7) |
| Hispanic | 257 (7.2) | 878 (8.3) | 202 (5.7) | 370 (8) | 1379 (10) | 219 (4.7) | 767 (5.9) | 1920 (4.9) | 308 (2.3) |
| Other/unknown | 537 (15.1) | 1864 (17.6) | 894 (25.3) | 466 (10.1) | 1432 (10.4) | 483 (10.3) | 1071 (8.2) | 3064 (7.8) | 774 (5.8) |
| Charlson-Deyo score |  |  |  |  |  |  |  |  |  |
| 0-1 | 2947 (82.8) | 8438 (79.6) | 2963 (84) | 4229 (91.4) | 12507 (90.8) | 4339 (92.8) | 11979 (91.4) | 35343 (90.5) | 12172 (91.6) |
| >2 | 614 (17.2) | 2169 (20.4) | 564 (16) | 400 (8.6) | 1263 (9.2) | 337 (7.2) | 1120 (8.6) | 3721 (9.5) | 1112 (8.4) |
| Insurance status |  |  |  |  |  |  |  |  |  |
| None | 118 (3.3) | 305 (2.9) | 55 (1.6) | 126 (2.7) | 420 (3.1) | 62 (1.3) | 376 (2.9) | 768 (2) | 198 (1.5) |
| Private | 1060 (29.8) | 3895 (36.7) | 1335 (37.9) | 1445 (31.2) | 4844 (35.2) | 1801 (38.5) | 4598 (35.1) | 14279 (36.6) | 5260 (39.6) |
| Medicare | 1950 (54.8) | 5099 (48.1) | 1433 (40.6) | 2679 (57.9) | 7287 (52.9) | 2427 (51.9) | 7048 (53.8) | 21262 (54.4) | 7064 (53.2) |
| Medicaid | 316 (8.9) | 996 (9.4) | 377 (10.7) | 273 (5.9) | 820 (6) | 226 (4.8) | 727 (5.6) | 1784 (4.6) | 384 (2.9) |
| Other/unknown | 117 (3.3) | 312 (2.9) | 327 (9.3) | 106 (2.3) | 399 (2.9) | 160 (3.4) | 350 (2.7) | 971 (2.5) | 378 (2.8) |
| Low education | 1647 (46.3) | 5064 (47.7) | 1732 (49.1) | 2104 (45.5) | 5979 (43.4) | 1716 (36.7) | 5682 (43.4) | 15731 (40.3) | 5031 (37.9) |
| Low income | 1404 (39.4) | 4109 (38.7) | 1271 (36) | 1829 (39.5) | 4813 (35) | 1559 (33.3) | 5126 (39.1) | 13440 (34.4) | 4473 (33.7) |
| Facility volume (median, IQR) | 0.2 (0.4) | 2.7 (3.3) | 12.9 (5) | 0.3 (0.4) | 1.9 (2.1) | 11.9 (6.3) | 0.7 (1.1) | 5.9 (6.7) | 36.2 (29) |
| Academic facilities, n (%) | 1480 (41.6) | 9153 (86.3) | 3366 (95.4) | 1492 (32.2) | 10148 (73.7) | 4676 (100) | 4225 (32.3) | 30152 (77.2) | 12518 (94.2) |
| Distance from facility (miles; median, IQR) | 7.5 (14.1) | 16.6 (39.9) | 27.3 (73.1) | 7.2 (13.3) | 14.3 (32) | 42.7 (82.5) | 8 (14.2) | 15.9 (34.2) | 42.2 (85) |
| Number of Facilities | 734 | 197 | 15 | 848 | 338 | 22 | 915 | 318 | 20 |

| **Supplementary Table 3.** Baseline clinical and demographic characteristics, stratified by RSMR tercile and tumor location. | | | | | | | | | |
| --- | --- | --- | --- | --- | --- | --- | --- | --- | --- |
| Characteristics | Liver | | | BTC | | | Pancreas | | |
|  | HPC | MPC | LPC | HPC | MPC | LPC | HPC | MPC | LPC |
| Total Patients | 9801 | 3685 | 4209 | 12298 | 4731 | 6046 | 37173 | 11925 | 16349 |
| Age (median, IQR) | 65 (14) | 65 (15) | 65 (15) | 67 (16) | 68 (17) | 68 (15) | 67 (14) | 67 (15) | 67 (15) |
| Male sex, n (%) | 6992 (71.34) | 2619 (71.07) | 3013 (71.58) | 6189 (50.33) | 2247 (47.5) | 2847 (47.09) | 18993 (51.09) | 6015 (50.44) | 8406 (51.42) |
| Race |  |  |  |  |  |  |  |  |  |
| White | 5655 (57.7) | 2358 (63.99) | 2459 (58.42) | 8977 (73) | 3540 (74.83) | 4305 (71.2) | 29704 (79.91) | 9547 (80.06) | 12393 (75.8) |
| Black | 1333 (13.6) | 552 (14.98) | 706 (16.77) | 880 (7.16) | 410 (8.67) | 614 (10.16) | 2977 (8.01) | 1110 (9.31) | 1812 (11.08) |
| Hispanic | 704 (7.18) | 235 (6.38) | 398 (9.46) | 1000 (8.13) | 390 (8.24) | 578 (9.56) | 1536 (4.13) | 526 (4.41) | 933 (5.71) |
| Other/Unknown | 2109 (21.52) | 540 (14.65) | 646 (15.35) | 1441 (11.72) | 391 (8.26) | 549 (9.08) | 2956 (7.95) | 742 (6.22) | 1211 (7.41) |
| Charlson-Deyo score |  |  |  |  |  |  |  |  |  |
| 0-1 | 7959 (81.21) | 2994 (81.25) | 3395 (80.66) | 11270 (91.64) | 4315 (91.21) | 5490 (90.8) | 33844 (91.04) | 10885 (91.28) | 14765 (90.31) |
| >2 | 1842 (18.79) | 691 (18.75) | 814 (19.34) | 1028 (8.36) | 416 (8.79) | 556 (9.2) | 3329 (8.96) | 1040 (8.72) | 1584 (9.69) |
| Insurance Status |  |  |  |  |  |  |  |  |  |
| None | 199 (2.03) | 123 (3.34) | 156 (3.71) | 247 (2.01) | 144 (3.04) | 217 (3.59) | 511 (1.37) | 311 (2.61) | 520 (3.18) |
| Private | 3709 (37.84) | 1198 (32.51) | 1383 (32.86) | 4624 (37.6) | 1527 (32.28) | 1939 (32.07) | 14404 (38.75) | 4164 (34.92) | 5569 (34.06) |
| Medicare | 4528 (46.2) | 1883 (51.1) | 2071 (49.2) | 6423 (52.23) | 2645 (55.91) | 3325 (55) | 19855 (53.41) | 6539 (54.83) | 8980 (54.93) |
| Medicaid | 905 (9.23) | 351 (9.53) | 433 (10.29) | 639 (5.2) | 300 (6.34) | 380 (6.29) | 1428 (3.84) | 646 (5.42) | 821 (5.02) |
| Other/unknown | 460 (4.69) | 130 (3.53) | 166 (3.94) | 365 (2.97) | 115 (2.43) | 185 (3.06) | 975 (2.62) | 265 (2.22) | 459 (2.81) |
| Low education | 4308 (43.95) | 1813 (49.2) | 2322 (55.17) | 4645 (37.77) | 2179 (46.06) | 2975 (49.21) | 13391 (36.02) | 5198 (43.59) | 7855 (48.05) |
| Low income | 3183 (32.48) | 1630 (44.23) | 1971 (46.83) | 3589 (29.18) | 1986 (41.98) | 2626 (43.43) | 10852 (29.19) | 4870 (40.84) | 7317 (44.76) |
| Facility volume (median, IQR) | 0.5 (2.07) | 0.2 (0.53) | 0.33 (0.8) | 0.67 (1.6) | 0.27 (0.57) | 0.6 (0.93) | 1.87 (5.32) | 0.6 (1.33) | 1.47 (2.73) |
| Academic facilities, n (%) | 8535 (87.08) | 2691 (73.03) | 2773 (65.88) | 9962 (81.01) | 3021 (63.86) | 3333 (55.13) | 29722 (79.96) | 7746 (64.96) | 9427 (57.66) |
| Distance from treating facility, (miles; median, IQR) | 17.4 (44.32) | 13.8 (39.75) | 11.4 (27.28) | 19.6 (47.88) | 12.2 (33.4) | 10.2 (21.08) | 20.3 (48) | 13.5 (34.4) | 11.6 (23.1) |
| Number of Facilities | 316 | 315 | 315 | 403 | 403 | 402 | 418 | 418 | 417 |

| **Supplementary Table 4**. Baseline clinical and demographic characteristics, stratified by reassignment status by volume-based reassignment and tumor location | | | | | | |
| --- | --- | --- | --- | --- | --- | --- |
| Characteristics | Liver | | BTC | | Pancreas | |
|  | Not reassigned | Reassigned | Not reassigned | Reassigned | Not reassigned | Reassigned |
| Total Patients | 3527 | 14168 | 4676 | 18399 | 13284 | 52163 |
| Age (median, IQR) | 64 (14) | 65 (15) | 67 (15) | 68 (15) | 67 (15) | 67 (14) |
| Male sex, n (%) | 2585 (73.3) | 10039 (70.9) | 2434 (52.1) | 8849 (48.1) | 6813 (51.3) | 26601 (51) |
| Race |  |  |  |  |  |  |
| White | 1989 (56.4) | 8483 (59.9) | 3661 (78.3) | 13161 (71.5) | 11185 (84.2) | 40459 (77.6) |
| Black | 442 (12.5) | 2149 (15.2) | 313 (6.7) | 1591 (8.6) | 1017 (7.7) | 4882 (9.4) |
| Hispanic | 202 (5.7) | 1135 (8) | 219 (4.7) | 1749 (9.5) | 308 (2.3) | 2687 (5.2) |
| Other/unknown | 894 (25.3) | 2401 (16.9) | 483 (10.3) | 1898 (10.3) | 774 (5.8) | 4135 (7.9) |
| Charlson-Deyo score |  |  |  |  |  |  |
| 0-1 | 2963 (84) | 11385 (80.4) | 4339 (92.8) | 16736 (91) | 12172 (91.6) | 47322 (90.7) |
| >2 | 564 (16) | 2783 (19.6) | 337 (7.2) | 1663 (9) | 1112 (8.4) | 4841 (9.3) |
| Insurance status |  |  |  |  |  |  |
| None | 55 (1.6) | 423 (3) | 62 (1.3) | 546 (3) | 198 (1.5) | 1144 (2.2) |
| Private | 1335 (37.9) | 4955 (35) | 1801 (38.5) | 6289 (34.2) | 5260 (39.6) | 18877 (36.2) |
| Medicare | 1433 (40.6) | 7049 (49.8) | 2427 (51.9) | 9966 (54.2) | 7064 (53.2) | 28310 (54.3) |
| Medicaid | 377 (10.7) | 1312 (9.3) | 226 (4.8) | 1093 (5.9) | 384 (2.9) | 2511 (4.8) |
| Other/Unknown | 327 (9.3) | 429 (3) | 160 (3.4) | 505 (2.7) | 378 (2.8) | 1321 (2.5) |
| Low education | 1732 (49.1) | 6711 (47.4) | 1716 (36.7) | 8083 (43.9) | 5031 (37.9) | 21413 (41.1) |
| Low income | 1271 (36) | 5513 (38.9) | 1559 (33.3) | 6642 (36.1) | 4473 (33.7) | 18566 (35.6) |
| Facility volume (median, IQR) | 12.9 (5.0) | 0.3 (0.9) | 11.9 (6.3) | 0.5(0.9) | 36.2 (29.0) | 1.1 (2.7) |
| Academic facilities, n (%) | 3366 (95.4) | 10633 (75) | 4676 (100) | 11640 (63.3) | 12518 (94.2) | 34377 (65.9) |
| Distance from facility (miles; median, IQR) | 27.3 (73.1) | 13.2 (32) | 42.7 (82.5) | 11.9 (26) | 42.2 (85) | 13.1 (27.8) |
| Number of Facilities | 15 | 931 | 22 | 1186 | 20 | 1233 |

| **Supplementary Table 5.** Baseline clinical and demographic characteristics, stratified by reassignment status by RSMR-based reassignment and tumor location | | | | | | |
| --- | --- | --- | --- | --- | --- | --- |
| Characteristics | Liver | | BTC | | Pancreas | |
|  | Not reassigned | Reassigned | Not reassigned | Reassigned | Not reassigned | Reassigned |
| Total Patients | 13486 | 4209 | 17029 | 6046 | 49098 | 16349 |
| Age (median, IQR) | 65 (14) | 65 (15) | 67 (16) | 68 (15) | 67 (14) | 67 (15) |
| Male sex, n (%) | 9611 (71.3) | 3013 (71.6) | 8436 (49.5) | 2847 (47.1) | 25008 (50.9) | 8406 (51.4) |
| Race |  |  |  |  |  |  |
| White | 8013 (59.4) | 2459 (58.4) | 12517 (73.5) | 4305 (71.2) | 39251 (79.9) | 12393 (75.8) |
| Black | 1885 (14) | 706 (16.8) | 1290 (7.6) | 614 (10.2) | 4087 (8.3) | 1812 (11.1) |
| Hispanic | 939 (7) | 398 (9.5) | 1390 (8.2) | 578 (9.6) | 2062 (4.2) | 933 (5.7) |
| Other/Unknown | 2649 (19.6) | 646 (15.3) | 1832 (10.8) | 549 (9.1) | 3698 (7.5) | 1211 (7.4) |
| Charlson-Deyo Score |  |  |  |  |  |  |
| 0-1 | 10953 (81.2) | 3395 (80.7) | 15585 (91.5) | 5490 (90.8) | 44729 (91.1) | 14765 (90.3) |
| >2 | 2533 (18.8) | 814 (19.3) | 1444 (8.5) | 556 (9.2) | 4369 (8.9) | 1584 (9.7) |
| Insurance Status |  |  |  |  |  |  |
| None | 322 (2.4) | 156 (3.7) | 391 (2.3) | 217 (3.6) | 822 (1.7) | 520 (3.2) |
| Private | 4907 (36.4) | 1383 (32.9) | 6151 (36.1) | 1939 (32.1) | 18568 (37.8) | 5569 (34.1) |
| Medicare | 6411 (47.5) | 2071 (49.2) | 9068 (53.3) | 3325 (55) | 26394 (53.8) | 8980 (54.9) |
| Medicaid | 1256 (9.3) | 433 (10.3) | 939 (5.5) | 380 (6.3) | 2074 (4.2) | 821 (5) |
| Other/Unknown | 590 (4.4) | 166 (3.9) | 480 (2.8) | 185 (3.1) | 1240 (2.5) | 459 (2.8) |
| Low education | 6121 (45.4) | 2322 (55.2) | 6824 (40.1) | 2975 (49.2) | 18589 (37.9) | 7855 (48) |
| Low income | 4813 (35.7) | 1971 (46.8) | 5575 (32.7) | 2626 (43.4) | 15722 (32) | 7317 (44.8) |
| Facility volume (median, IQR) | 0.3 (1) | 0.3 (0.8) | 0.4 (0.9) | 0.6 (0.9) | 1.07 (3.0) | 1.47 (2.7) |
| Academic facilities, n (%) | 11226 (83.2) | 2773 (65.9) | 12983 (76.2) | 3333 (55.1) | 37468 (76.3) | 9427 (57.7) |
| Distance from treating facility, (miles; median, IQR) | 16.5 (42.8) | 11.4 (27.3) | 17.4 (43.2) | 10.2 (21.1) | 18.6 (44.2) | 11.6 (23.1) |
| Number of Facilities | 631 | 315 | 806 | 402 | 836 | 417 |
