# Supplementary figures and images for: Comparison of Hospital Volume and Risk-Standardized Mortality Rate as a Proxy for Hospital Quality in Complex Oncologic Hepatopancreatobiliary Surgery
Source: Ann Surg Oncol. 2024 May 3;31(8):4922–30. doi: 10.1245/s10434-024-15361-2 (PMC11236847; doi:10.1245/s10434-024-15361-2)

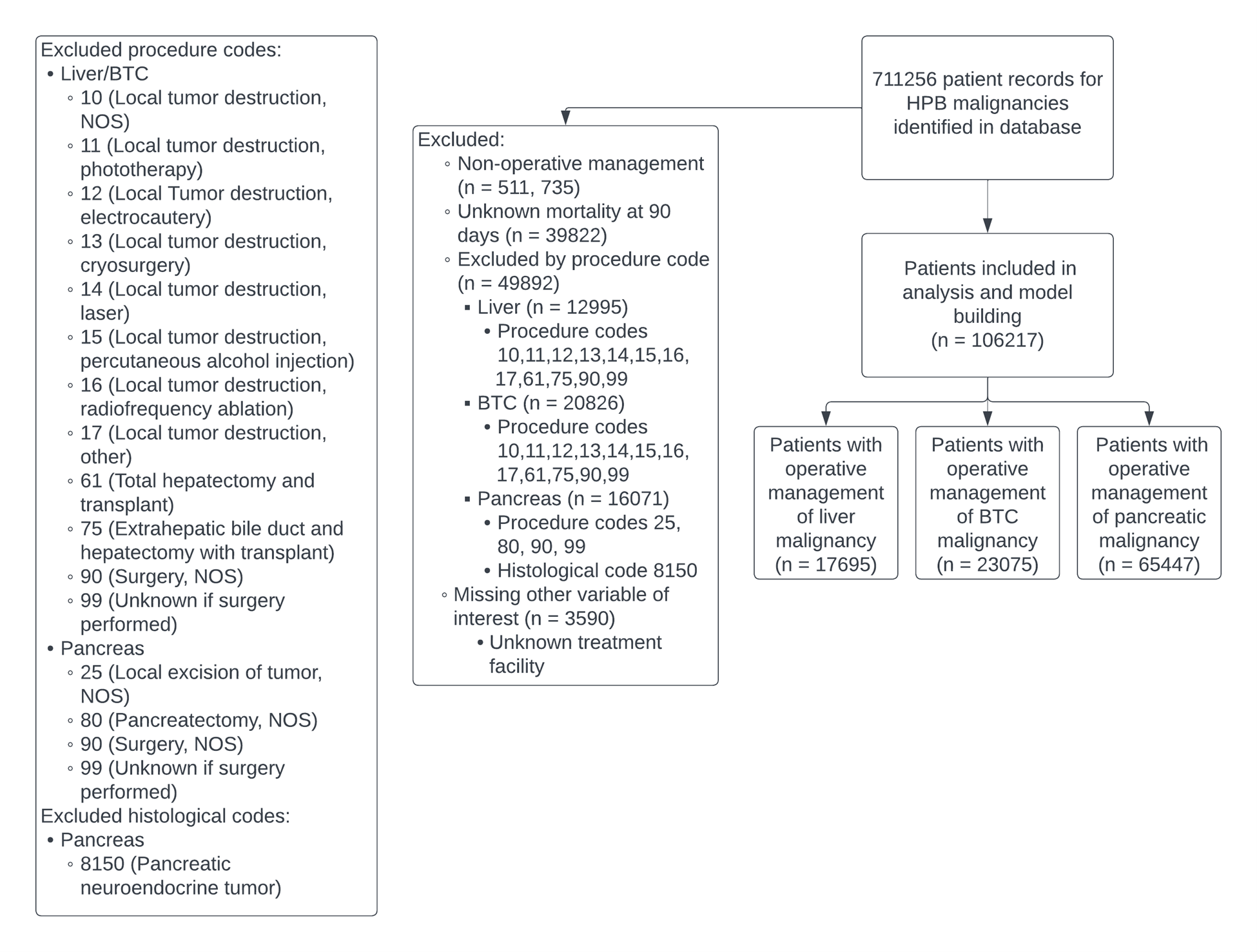

Supplement: Supplementary file 2 — Supplementary file2 (TIF 1246 kb) [file 10434_2024_15361_MOESM2_ESM.tif]

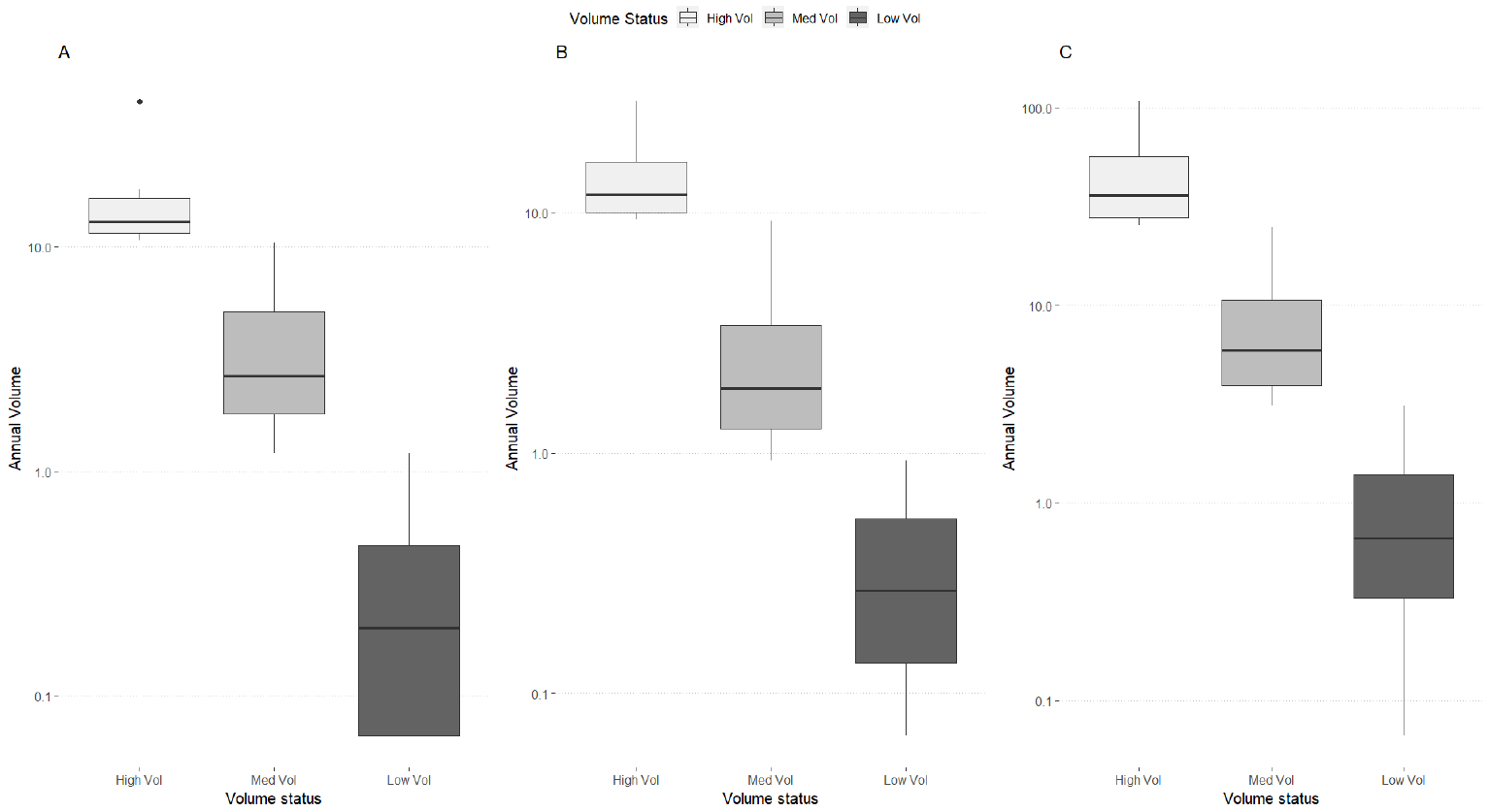

Supplement: Supplementary file 3 — Supplementary file3 (TIF 44 kb) [file 10434_2024_15361_MOESM3_ESM.tif]

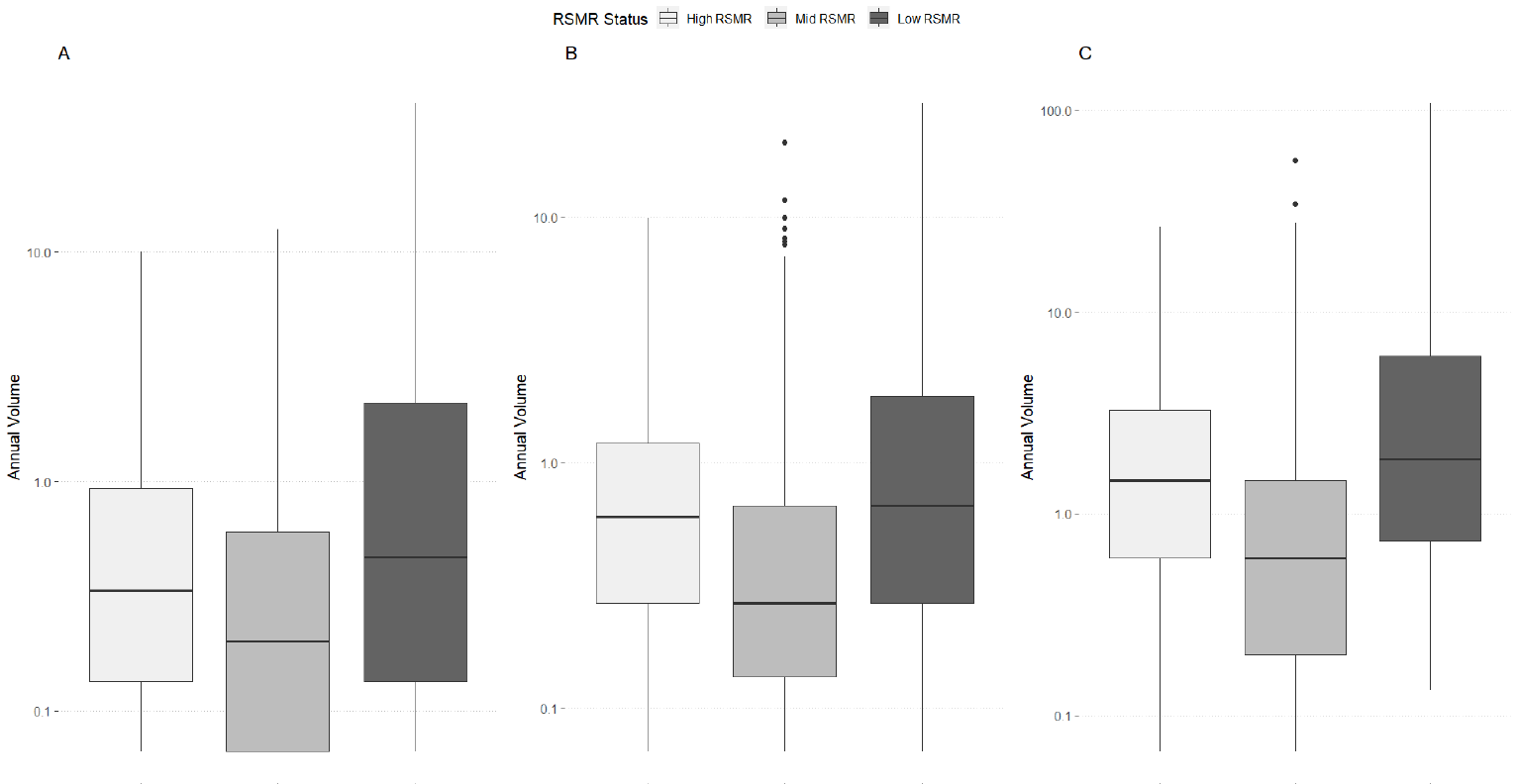

Supplement: Supplementary file 4 — Supplementary file4 (TIF 55 kb) [file 10434_2024_15361_MOESM4_ESM.tif]

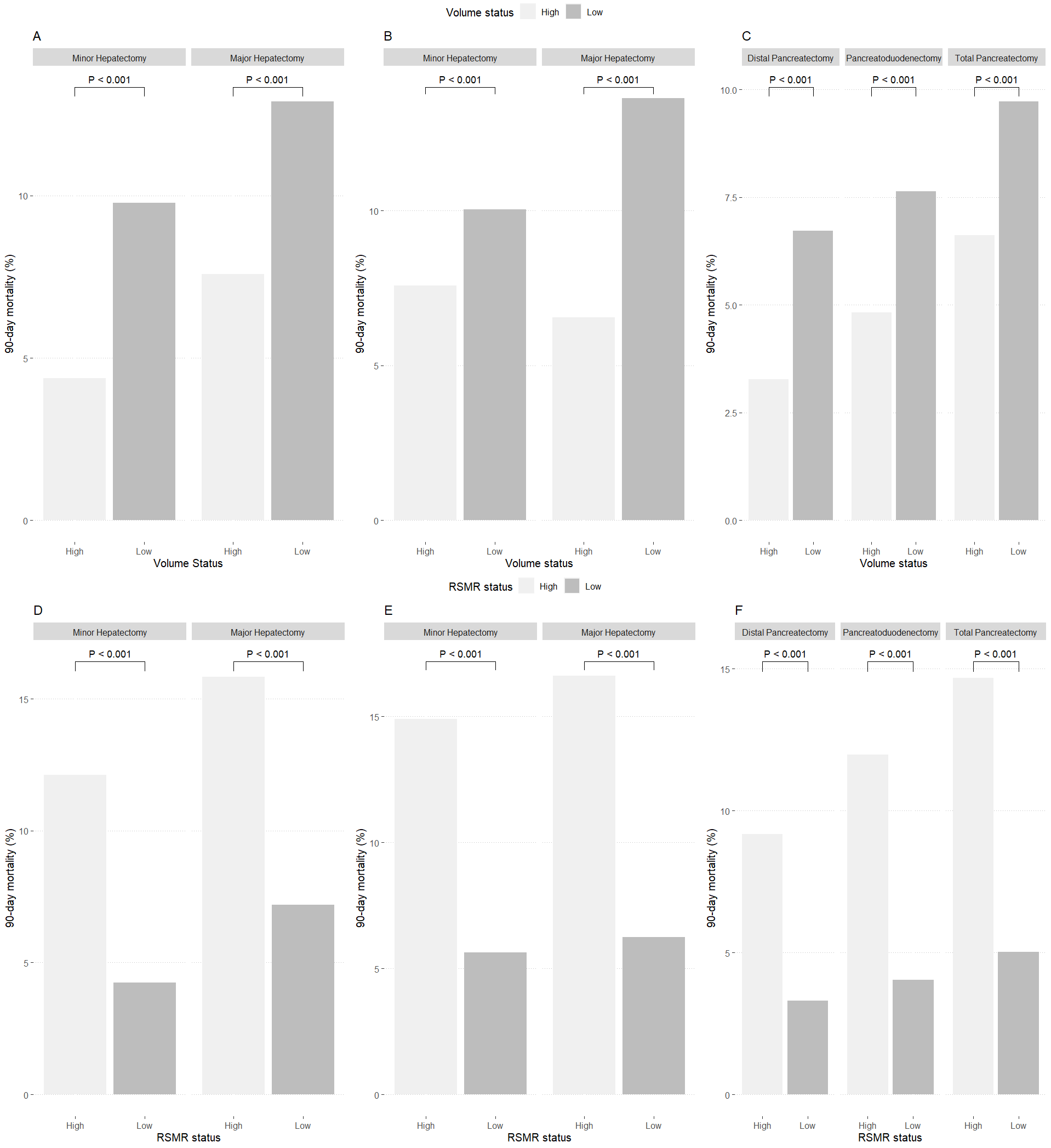

Supplement: Supplementary file 5 — Supplementary file5 (TIF 465 kb) [file 10434_2024_15361_MOESM5_ESM.tif]
